# Protocol for a systematic evaluation of pediatric pharmacy development and pediatric pharmacy experts’ research area in China

**DOI:** 10.1097/MD.0000000000013597

**Published:** 2018-12-14

**Authors:** Hao Li, Shi-Ying Huang, Shun-Guo Zhang, Min-Ling Chen, Zhi-Chun Gu, Fang-Hong Shi

**Affiliations:** a Department of Pharmacy, Shanghai Children's Medical Center, Shanghai Jiao Tong University School of Medicine, Shanghai; b Department of Pharmacy, Renji Hospital, School of Medicine, Shanghai Jiaotong University, Shanghai, P.R. China.

**Keywords:** expert, pediatric, pediatric pharmacy development, pharmacy, research area

## Abstract

**Background:**

The pediatric pharmacy research status of children's hospitals in China is still unknown. Our previous findings suggest the regional differences in academic level in tertiary (grade III level A) children's hospitals in China.

**Methods:**

This systemic evaluation described in this protocol will be conducted to follow the Cochrane Handbook. We will perform a systemic literature search of relevant databases including Chinese databases (CNKI, Wanfang Data, VIP Paper Check System) and English databases (Medline, EMbase, Cochrane Library) from inception to December 31, 2018. The search strategy will be enacted according to the guidance offered from the Cochrane Handbook. Two rounds of searches will be conducted to prevent the omission of relevant literature. A pre-set grading standard will be used to give calculation weight (*W*) to evaluate the quality of each article. Data synthesis will be performed using STATA software (version 13.1, Statacorp, College Station, Texas). Pediatric pharmacy development index (*PPDI*) of each hospital will be used to evaluate the pediatric pharmacy development in each tertiary children's hospitals. The cumulative calculation weight (∑W) and annual calculation weight (∑_year_W) will be used to evaluate the academic level of pharmaceutical departments in different tertiary children's hospitals. Subgroup analysis will be performed to compare the number of different types of articles published between different hospitals base on different research areas such as policy research, basic research, and clinical research.

**Results:**

In this article, we will evaluate pediatric pharmacy development and the research area of pediatric pharmacy experts in China. Based on the results from this research, we will analyze the professional backgrounds of pediatric pharmacy experts from 23 tertiary children's hospitals in China. According to the contents and research directions of literature published by the pediatric pharmacy experts in these 23 hospitals, we will determine the professional field of pediatric pharmacy experts and establish an expert database. In the process of formulating the related national or local policies in the future, the expert database will be selected accurately to reach the expert consensus.

**Conclusion:**

Our study will provide a comprehensive picture of pediatric pharmacy development in China. The pediatrics pharmacy expert's database constructed by this study will be used to build consensus on pediatric pharmacology in the future.

## 1 Introduction

In 1997, both the European Commission and the US Food and Drug Administration (FDA) took the initiative by introducing incentives for best pediatric pharmaceuticals development, with the adoption of the FDA Modernization Act.^[1]^ The Best Pharmaceuticals for Children Act in 2002 (BPCA 2002) and Pediatric Research Equity Act in 2003 made pediatric studies mandatory for those new products that would be used in pediatric patients and added incentives for pharmaceutical companies by extending of market exclusivity and/or patent. However, there is still exist a huge absence of specific pediatric medicines. In China, this huge demand of pediatric best pharmaceuticals is met by extemporaneously prepared products to “Make Medicine Child Size” from tablets or capsules.^[2–4]^ Such preparations are a threat to quality, efficacy, and safety of formal drugs. Up to this day, the status of pediatric pharmacy development in Chinese tertiary children's hospitals is still unknown. Our previous findings suggest that there are regional differences in academic level in tertiary children's hospitals in China.^[5]^ In recent years, academic papers are important reference indicators for evaluating the development and capabilities of disciplines in Chinese academic assessment. In this study, we will evaluate the development of pediatric pharmacy in China based on the publications published by each department of pharmacy in each Chinese tertiary children's hospital. Furthermore, we will analyze the professional backgrounds of pediatric pharmacy experts from 23 tertiary children's hospitals in China. A pediatric experts’ database will be built based on the professional field resulted from publications. This database may help to the expert selection in the process of formulating the related national or local policies in the future.

## 2 Methods

### 2.1 Registration

The reporting of the research will follow the principle of the Cochrane Handbook and conduct following a priori established protocol (PROSPERO: CRD42018103083). Ethical approval is not required because this is a literature-based study.

### 2.2 Criteria for considering studies for this study

All literature will be used for the data analysis including, but not limited to, research papers, reviews, case reports, opinions, etc. If the first affiliation of the first author or correspondence author is not from department of pharmacy in the analyzed tertiary children's hospital in China, the article will be excluded in this study.

### 2.3 Types of outcome measures

This protocol proposes to assess the pediatric pharmacy development index (PPDI) of each tertiary children's hospital in China.

### 2.4 Search methods for identification of studies

**Figure 1:**
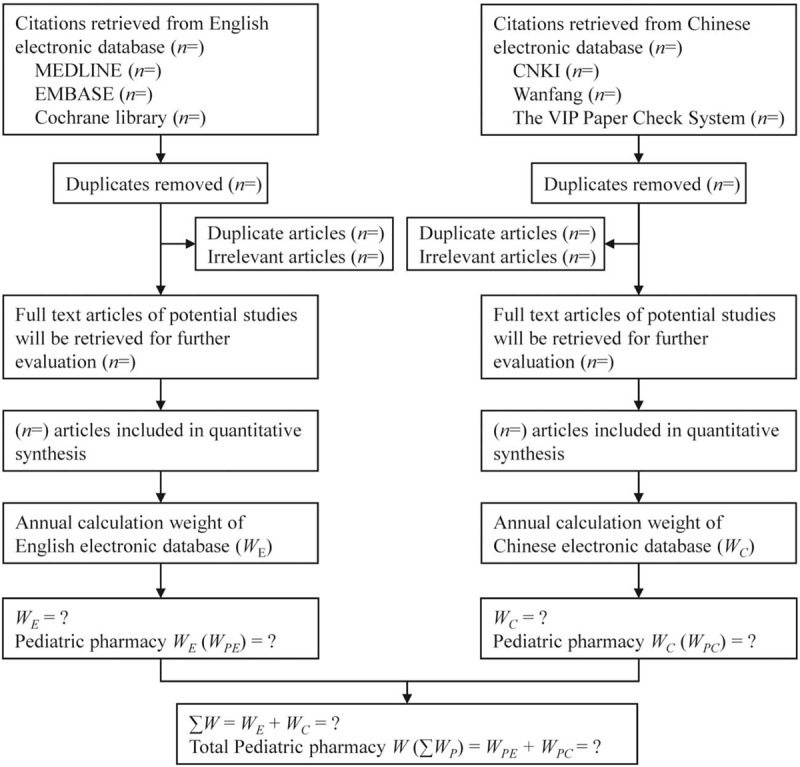
Study follow chart.

We will perform a comprehensive and systematic literature search of relevant databases including Chinese databases (CNKI, Wanfang, and the VIP Paper Check System) and English databases (MEDLINE, EMbase and The Cochrane Library) from their inception dates to December 31, 2018. The search strategy will be conducted in accordance with the guidance set out in the Cochrane Handbook. We will conduct 2 rounds of searches to prevent the omission of relevant literature. In the first round, we will search the articles according to the name of the affiliation. Briefly, the search terms will include “department of pharmacy [Affiliation]” and the name of each tertiary children's hospital in China (Table 1). Furthermore, we will perform a fuzzy search for the non-standard affiliation names of each tertiary children's hospital. HL and S-YH will select and confirm all the publication most relevant to our study. Lead author's and corresponding author's information will be collected. In the second round of search, lead author's and corresponding author's information will be used to verify the literature. Any disagreements will be resolved by consensus, or by consulting a third author (S-GZ). The details of the selection process are shown in Figure 1.

**Table 1:**
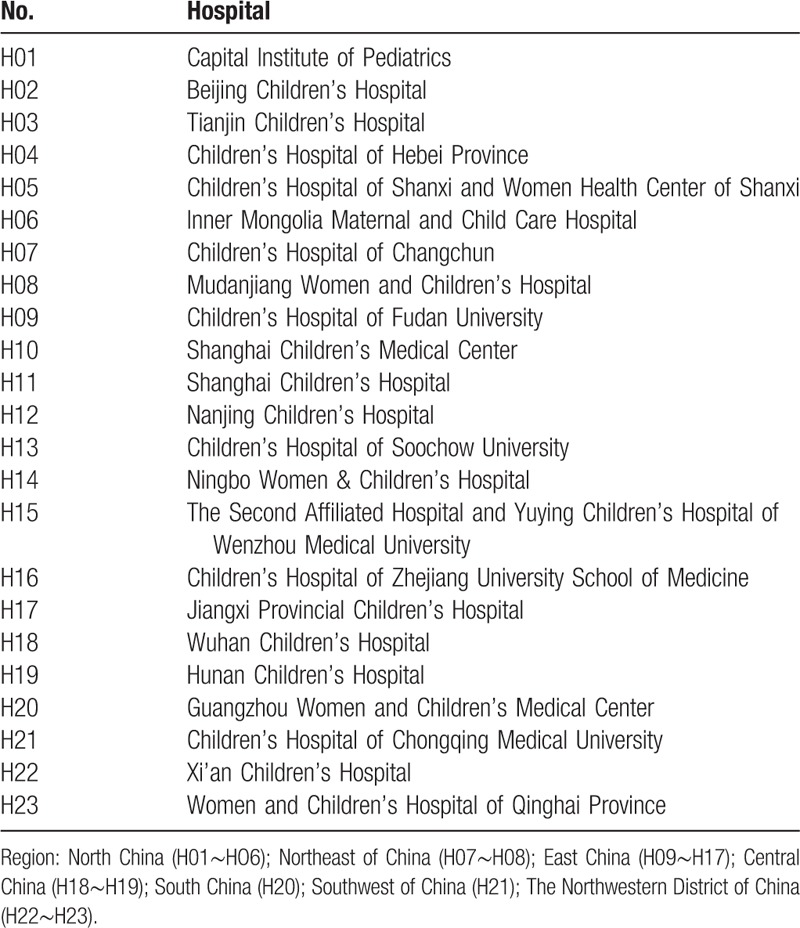
Hospital name of 23 tertiary children's hospitals.

### 2.5 Data analysis

Two authors (HL and S-YH) will independently analysis data and any disagreements are resolved by consensus or consulting a third author (S-GZ). Literature that are not conformed to the established inclusion criteria will be excluded. PPDI will be used to evaluate pediatric pharmacy development of each tertiary children's hospital. The quantity and quality of publications will be used to evaluate the academic level of each hospital. English papers will be judged according to whether they are indexed in Science Citation Index (SCI) and magazine impact factors. Chinese papers will be evaluated according to whether the magazine is indexed in Chinese Scientific and Technical Papers and Citations (CSTPC). The cumulative calculation weight (*∑W*) and annual calculation weight (*W*_E_ + *W*_*C*_) will be used to evaluate the academic level of pharmaceutical departments in different tertiary children's hospitals. In addition, pediatric pharmacy development will be evaluated whether the content of the publication is related to pediatric pharmacy by using pediatric pharmacy calculation weight (*W*_p_). The specific weighting coefficients are listed in Table 2.

*W* = (A_F_ + A_C_) × T × J
*W*_p_ = P × (A_F_ + A_C_) × T × J
*PPDI* = ∑*W*_*P*_ = *W*_*PE*_ *+* *W*_*PC*_

**Table 2:**
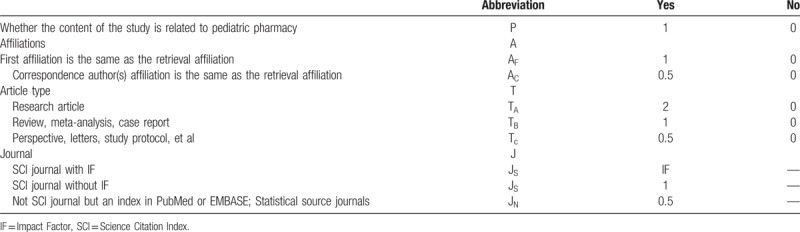
Weighting coefficients.

### 2.6 Subgroup analysis

We plan to perform subgroup analyses based on different types of articles published between different hospitals. The content of the subgroup analysis mainly includes the strength of policy research, the strength of basic research, and the strength of clinical research. The strength of real-world research will be analyzed in the strength analysis of clinical research. Pediatric pharmacy calculation weight (*W*_p_) of different types of articles will be used to evaluate the strength of different research ability.

### 2.7 Pediatric experts’ database

Electronic database will be developed, the substantial contents of each selected articles will be extracted by 2 authors (HL and S-YH) respectively and the following data will be extracted: the name of the first author, the name of the co-first author(s), the name of the correspondence author(s), authors’ affiliations, telephone numbers, and E-mails. Enlargement of pediatric pharmacy experts will be completed by S-GZ and M-LC.

### 2.8 Statistical analyses

Analyses will be performed by Stata V13.1 (STATA Corp, College Station, TX).

### 2.9 Ethics and dissemination

This systematic analysis does not require ethical assessment because only indirect literature will be included and evaluated.

## 3 Discussion

The absence of specific pediatric medicines leads to a serious threat to efficacy and safety in children's hospital. In the US, the FDA Modernization Act of 1997, BPCA in 2002, Pediatric Research Equity in 2003 and FDA Amendments Act of 2007 encouraged the pediatric drug development.^[1]^ Similarly, in Europe, the European Commission also took the initiative in 1997 and implemented pediatric regulation governing development and authorization of medicines for pediatric use in 2007.^[1]^ These acts encouraged pharmaceutical companies to perform pediatric clinical studies on new drug products.^[6]^ However, there are quite few new drugs approved for pediatric use in China. Up to now, there is no such act encouraging pharmaceutical companies to develop pediatric suitable drug products.

Before the analysis of the current development situation of pediatric and pediatric pharmacy in China, it is unclear how to improve pediatric medication status. Our previous study suggested there were obvious regional differences in the development of pediatric pharmacy in China.^[5]^ In addition, “Make Medicine Child Size” are very common in Chinese children's hospitals. In order to improve the current situation of pediatric medication and promote rational medication use for children, we will systematically evaluate Chinese pediatric pharmacy development and pediatric pharmacy experts’ research area in China. To the best of our knowledge, there were 23 tertiary children's hospitals in China. In recent years, academic publications are important reference indicators for evaluating the development and academic capabilities of hospitals. This study will be based on an analysis of publications from department of pharmacy in Chinese tertiary children's hospitals. We will build a pediatric expert database based on author information obtained from publications. Professional backgrounds of each pediatric pharmacy expert from 23 tertiary children's hospital will be analyzed. Professional field of each expert will be determined and divided based on the research area and calculation weight of each article. In the future, the screening of pediatric pharmacy experts to build an expert consensus on pediatric related national or local policies will be derived from the database constructed in this study.

### 3.1 Strengths and limitations of this study

This is the first comprehensive evaluation of pediatric pharmacy development and pediatric pharmacy area in China.

The results of this systematic analysis will help to understand pediatric pharmacy development in China.

The pediatric pharmacy experts’ database constructed in this study will help for the screening of pediatric pharmacy experts based on their research area.

The methods of this study are state of rigorous, including comprehensive literature search, explicit inclusion and exclusion criteria, independent study selection, data analysis, and statistical analysis.

We limit our analyses to not include the quantity and quality of grant of each department of pharmacy in each tertiary children's hospital.

We also limit our study to not include in the pediatrics of comprehensive tertiary hospitals, although some comprehensive tertiary hospitals have excellent pediatrics, such as the department of pediatrics of Xinhua Hospital, which also affiliated to Shanghai Jiao Tong University School of Medicine.

We also limit our study to not include maternal and infant hospitals. We do not include these hospitals as pharmaceutical departments in these hospitals are not only responsible for medication for children, but also for medication for adults, which may lead to bias in our study.

## Author contributions

HL, S-YH, and S-GZ exacted and analyzed the data and wrote the first draft of the protocol, and Z-CG helped with the design of the protocol and HL submitted the registration on PROSPERO. S-GZ and M-LC enlarged pediatric pharmacy experts database. Z-CG and F-HS provide professional support of this article Z-CG revised the manuscript. Z-CG is the guarantors for the publication and takes the responsibility for the paper. All authors participated in read and approved the final manuscript.

**Competing interests:** None declared.

**Conceptualization:** Hao Li, Shi-Ying Huang, Shun-Guo Zhang, Min-Ling Chen, Zhichun Gu, Fang-Hong Shi.

**Conceptualization:** Zhi-Chun Gu, Fang-Hong Shi

**Data curation:** Hao Li, Shi-Ying Huang, Shun-Guo Zhang.

**Patient consent:** Not required.
